# Crystallography of a Lewis-Binding Norovirus, Elucidation of Strain-Specificity to the Polymorphic Human Histo-Blood Group Antigens

**DOI:** 10.1371/journal.ppat.1002152

**Published:** 2011-07-21

**Authors:** Yutao Chen, Ming Tan, Ming Xia, Ning Hao, Xuejun C. Zhang, Pengwei Huang, Xi Jiang, Xuemei Li, Zihe Rao

**Affiliations:** 1 National Laboratory of Biomacromolecules, Institute of Biophysics, Chinese Academy of Sciences, Beijing, China; 2 Division of Infectious Diseases, Cincinnati Children's Hospital Medical Center, Cincinnati, Ohio, United States of America; 3 University of Cincinnati College of Medicine, Cincinnati, Ohio, United States of America; North Carolina State University, United States of America

## Abstract

Noroviruses, an important cause of acute gastroenteritis in humans, recognize the histo-blood group antigens (HBGAs) as host susceptible factors in a strain-specific manner. The crystal structures of the HBGA-binding interfaces of two A/B/H-binding noroviruses, the prototype Norwalk virus (GI.1) and a predominant GII.4 strain (VA387), have been elucidated. In this study we determined the crystal structures of the P domain protein of the first Lewis-binding norovirus (VA207, GII.9) that has a distinct binding property from those of Norwalk virus and VA387. Co-crystallization of the VA207 P dimer with Le^y^ or sialyl Le^x^ tetrasaccharides showed that VA207 interacts with these antigens through a common site found on the VA387 P protein which is highly conserved among most GII noroviruses. However, the HBGA-binding site of VA207 targeted at the Lewis antigens through the α-1, 3 fucose (the Lewis epitope) as major and the β-N-acetyl glucosamine of the precursor as minor interacting sites. This completely differs from the binding mode of VA387 and Norwalk virus that target at the secretor epitopes. Binding pocket of VA207 is formed by seven amino acids, of which five residues build up the core structure that is essential for the basic binding function, while the other two are involved in strain-specificity. Our results elucidate for the first time the genetic and structural basis of strain-specificity by a direct comparison of two genetically related noroviruses in their interaction with different HBGAs. The results provide insight into the complex interaction between the diverse noroviruses and the polymorphic HBGAs and highlight the role of human HBGA as a critical factor in norovirus evolution.

## Author Summary

The interactions of noroviruses with histo-blood group antigens (HBGAs) are diverse, in which strains in both genogroups I and II (GI and GII) recognizing either the secretor or the non-secretor (Lewis) HBGAs have been reported. The crystal structures of the HBGA binding interfaces of two secretor binders (Norwalk virus, GI.1 and VA387, GII.4) have been elucidated. In this study we determined the crystal structure of the HBGA-binding interface of the first Lewis-binder (VA207, GII.9) and compared it with those of the two secretor binders. VA207 binds to the Lewis antigens via the Lewis epitope (α-1, 3 fucose) as the major interacting residue, which is distinct from the two secretor binders that interact with the secretor antigens through the A or H epitope as a major interacting residue. In addition, precursor saccharide was involved in binding and has a role in strain-specificity of VA207. VA207 shares a conserved HBGA binding interfaces with VA387, suggesting a strong selection of human HBGAs in norovirus evolution. The distinct binding modes between these two GII strains suggest a potential host-driving force on the diversity of noroviruses by the polymorphic HBGAs. The crystal structures resolved in this study also would facilitate the antiviral drug design against noroviruses.

## Introduction

Noroviruses, a genus in the family *Caliciviridae,* are a group of small-round structured, nonenveloped RNA viruses causing epidemic acute gastroenteritis in all age in both developing and developed countries [1], [2], [3]. Structurally, norovirus possesses an outer protein capsid that encapsulates a single-stranded, positive sense RNA genome of ∼7.5 kb. Norovirus capsid displays an icosahedral symmetry that is composed of 180 copies of a single major structural protein, the capsid protein (VP1), which organize into 90 dimers [4]. Each VP1 can be divided into two major domains, the N-terminal shell (S) and the C-terminal protruding (P) domains, linked by a short hinge. The S domain forms the interior shell; the P domain constitutes the arch-like P dimer of the capsid, while the hinge provides flexibility between the two major domains [4]. The P domain is further divided into P1 and P2 subdomains, forming the leg and the head of the protruding P dimer, respectively [4].

*In vitro* expression of full-length VP1 assembles empty virus-like particles (VLPs) that are structurally and antigenically indistinguishable from the authentic virus. The S and the P domains were found to be structurally and functionally independent. Expression of the S domain alone formed smooth, thin-layer S particles without the protruding structures [5], [6], while production of P domain *in vitro* resulted in P dimer, P particle, and small P particle [6], [7], [8], [9], [10], [11], [12], [13]. Functional analyses showed that the P dimer and the P particle, but not the S particle, bind to histo-blood group antigens (HBGAs), the host susceptible factors of noroviruses (see below), indicating that the P domain is the HBGA binding domain [3], [6], [8], [13], [14]. Since human noroviruses are not cultivatable in the laboratory, these VLPs and subviral particles are valuable tools for studying the virus-host interaction and as vaccine candidates for noroviruses. The P particles are particularly useful due to their simple procedures and high yields of production in a bacterial system.

Noroviruses recognize the HBGAs as important host susceptible factors [3], [14], [15]. HBGAs are the determinants of blood types that are complex carbohydrates presenting at the outermost ends of N- or O-linked glycans or glycolipids [16], [17] on the mucosal epithelia of intestinal and genitourinary tracts. They are also present as free oligosaccharides in biologic fluids such as blood, saliva and milk. Noroviruses interact with HBGAs in a strain-specific manner, in which all common HBGAs such as A, B, H, and Lewis antigens are involved in norovirus recognition. The carbohydrate binding interfaces of noroviruses have been elucidated by crystallography [18], [19], [20] followed by mutagenesis studies [10], [11], [21]. They are located at the distal end of the arch-like P dimer, corresponding to the outermost surface of the viral capsid.

The HBGA-binding sites of two noroviruses, Norwalk virus (GI.1) and VA387 (GII.4), each representing a major genogroup (GI and GII) of human noroviruses, have been elucidated [18], [19], [20]. The HBGA-binding sites of the two strains differ in their precise locations, structures, sequence compositions and binding modes to carbohydrates, although they share the same targets of A and H antigens [22], [23]. Further study showed that the HBGA-binding sites are conserved within but not between the two major genogroups of human noroviruses [11], [19], [20], although the two genogroups share the same ligand repertoire of human HBGAs. These data indicate that the HBGA binding is a prerequisite of norovirus infection and therefore important in the evolution of human noroviruses [11].

It has been noted for Norwalk virus (GI.1) that the same HBGA-binding interface can interact with different carbohydrate antigens by distinct bonds. For example, in the interaction with the type A trisaccharides, the α-GalNAc is the major contact, while in the case of the H pentasaccharide, both the β-Gal and the α-1, 2 Fuc are the major contacts [18], [20]. On the other hand, VA387 (GII.4) recognizes both the A and B trisaccarides through the same α-1, 2 Fuc as major contact [19]. These data indicate a great flexibility in the interaction between noroviruses and HBGAs, in which the same binding interface can bind to different HBGAs (Norwalk to A/H and VA387 to A/B antigen), while distinct binding interfaces are able to bind the same HBGAs (Norwalk and VA387 to A antigen). This structural information helps the explanation of the complex virus-host interaction and evolution of noroviruses.

Both Norwalk virus and VA387 [3], [14] recognize the secretor antigens (A/B/H), but not the non-secretor antigens. In this study, we performed a crystallographic study on a first non-secretor binding strain (VA207, GII.9) [23]. Our data indicated that VA207 uses the common genogroup II HBGA binding interface described for VA387 but interacts with a different set of HBGAs (Le^y^ and sialyl Le^x^) through a completely different binding mode. Instead of targeting at the secretor epitopes of the HBGAs in secretor binders (VA387 and Norwalk virus), VA207 targets at the non-secretor (Lewis) epitope (the α-1, 3 fucose) as the major contact. Mutagenesis study identified amino acids that are responsible for the type-specificity of VA207. These data for the first time elucidate the genetic and structural basis of strain-specificity by a direct comparison of two genetically related noroviruses in their interaction with different HBGAs, highlighting the role of human HBGA as a critical factor in norovirus evolution. The structural data would help antiviral development for disease control and prevention of noroviruses.

## Materials and Methods

### Expression and purification of recombinant P protein for crystallization

The cDNA fragment encoding P domain of norovirus VA207 (GII.9) (amino acid 222–537, GenBank accession#: AY038599) was cloned into the expression vector pGEX-6P-1 (GE Healthcare Life Sciences) and was expressed as a GST fusion protein in *E. coli* BL21 DE3 at 16°C overnight induced with 0.5 mM Isopropyl β-D-1-Thiogalactopyranoside (IPTG). The recombinant protein was purified using glutathione-sepharose 4B (GE Healthcare Life Sciences) according to the manufacturer's protocol. The P protein was cleaved from the GST tag with Prescission Protease (GE Healthcare Life Sciences) at 4°C overnight. The eluted P protein was confirmed by Mass Spectrometry Fingerprint to bind to *E. coli* chaperone GroEL, which could be further separated from P protein by Mono Q anion ion exchange (GE Healthcare Life Sciences) at pH 7.3. P protein was eluted at ∼300 mM NaCl while GroEL was eluted at ∼500 mM NaCl. P protein was then dialysed against 20 mM HEPES (pH 7.3), 150 mM NaCl before crystallization.

### Production of P particle for mutagenesis study

The expression construct of wild type P particles of VA207 were generated previously [11] by cloning the P protein-encoding sequences into the plasmid pGEX-4T-1 (GE Healthcare Life Sciences). Mutant P particles with single amino acid mutation at the HBGA binding interface were constructed by site-directed mutagenesis using the wild type constructs as templates. Site-directed mutagenesis was conducted using the QuickChange Site-Directed Mutagenesis Kit (Stratagene, La Jolla, CA) using following primer pairs: caggtgacgccacggcggcccatgaggcaag/cttgcctcatgggccgccgtggcgtcacctg (R346A), ctcaacctcaagcgcttttgaaacaaacc/ggtttgtttcaaaagcgcttgaggttgag (D374A), ccaataggtatcgccattgagggcaattct/agaattgccctcaatggcgatacctattgg(Y389A), ccaggagctagtgcccacacaaatggg/cccatttgtgtgggcactagctcctgg (G440A), ccaggagctagtggcgccacaaatggggagatg/catctccccatttgtggcgccactagctcctgg (H441A), ttcatcccaggagctgctggccacacaaatgg/ccatttgtgtggccagcagctcctgggatgaa (S439A), as described previously [6], [10], [11], [21]. The wild type and mutant P particles were expressed in *E. coli* BL21 DE3 and then purified using the Glutathione Sepharose 4 flow (GE Healthcare Life Sciences, Piscataway, NJ) according to the manufacturer's protocol as described elsewhere [6], [8], [9], [10], [11]. The P proteins were released from GST tag by thrombin (GE Healthcare Life Sciences) digestion. The formation of P particle was determined by gel filtration using a size-exclusion column Superdex 200 (GE Healthcare Life Sciences) powered by an AKTA-FPLC system (model 920, GE Healthcare Life Sciences) followed by SDS-PAGE electrophoresis, in which the P particles formed a peak at ∼830 kDa. None of the designed single residue mutations in this study affected P particle formation and none of the mutants revealed any detectable reduction in reactivity to the hyperimmune serum against VLP compared with that of the wild type P particle.

### Crystallization of P protein and its complexes with Le^y^ and sialyl Le^x^ tetrasaccharides

**Figure 1:**
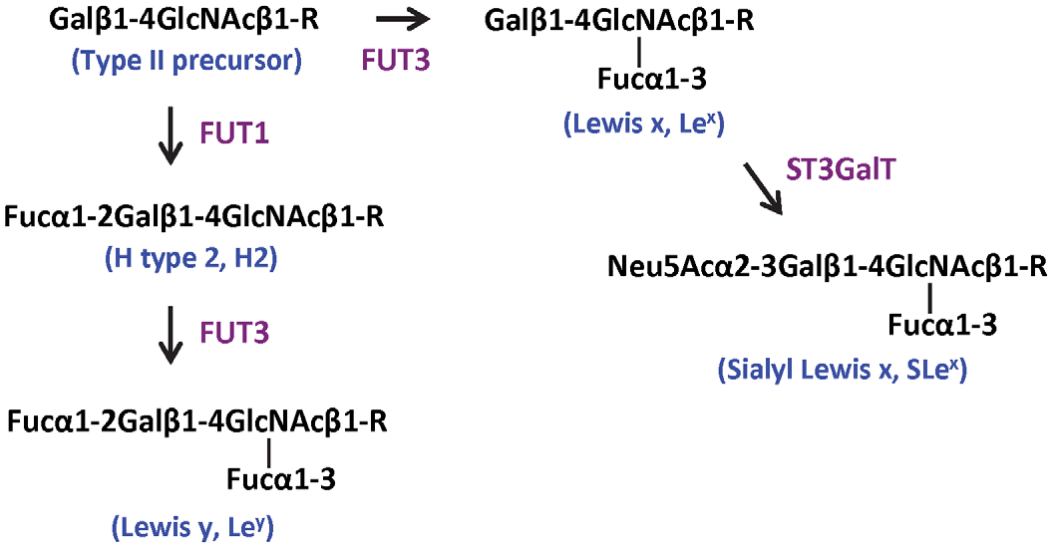
Biosynthesis pathways of Lewis y and sialyl Lewis x starting from the type II precursor (Galβ1–4GlcNAcβ1-R). The sequences, structures, linkages, and nomenclatures of the carbohydrates, as well as catalytic enzymes are indicated. Gal, galactose; GlcNAc, N-acetylglucosamine; Fuc, fucose; Neu5Ac, N-acetylneuraminic acid (sialic acid); FUT1, α-1, 2 fucosyltransferase; FUT3, α-1, 3/4 fucosyltransferase; ST3GalT, α-2,3 sialyltransferase; R represents a scaffold that might be a glycolipid or a glycoprotein.

P protein was concentrated to 12 mg/ml. Native crystals were grown using the hanging drop vapor diffusion method by mixing 1.5 µl protein solution with an equal volume of reservoir solution containing 12% (w/v) polyethylene glycol (PEG) 3350 and 50 mM magnesium formate. For growth of complex crystals, the two tetrasaccharides, Lewis y {α-Fuc-(1→2)-β-Gal-(1→4)-[α-Fuc-(1→3)]-GlcNAc, Le^y^} and Sialyl Lewis x {α-NeuNAc-(2→3)-β-Gal-(1→4)[α-Fuc-(1→3)]-GlcNAc, SLe^x^} (Sigma) were first separately dissolved in ddH_2_O to a concentration of 0.3 mM and mixed with P protein to final molar ratio of 60∼100∶1. The mixtures were incubated at 4°C for 2 h before mixed with reservoir solution containing 10–15% (w/v) PEG 3350 and 50 mM magnesium formate. The hanging drops were equilibrated over 500 µl reservoir solution, and crystals could be harvested in two weeks. The biosynthesis pathways of Le^y^ and SLe^x^ are shown in Figure 1 with indications of sequences, structures, linkages, and nomenclatures.

### Data collection and processing

X-ray diffraction data were collected from flash-cooled crystals using an in-house Rigaku FR-E rotating anode X-ray generator with an R-AXIS IV++ detector at a wavelength of 1.5418 Å for native P protein crystals and P protein complexed with Le^y^ tetrasaccharide crystals. Diffraction data for P protein complexed with SLe^x^ tetrasaccharide crystals were collected at BL17A of the KEK Photon Factory (Japan) at a wavelength of 1.0000 Å. The cryoprotectant solution contained 12% (w/v) PEG 3350, 50 mM magnesium formate and 15% (v/v) glycerol. The data were processed, scaled and merged using the HKL2000 program package [24]. Statistics for data collection and processing are summarized in Table 1.

**Table 1:** Data collection statistics.

Parameters		Native P protein	Complex with Le^y^ tetrasaccharide	Complex with SLe^x^ tetrasaccharide
Spacegroup		P2_1_2_1_2	P2_1_2_1_2_1_	P2_1_2_1_2_1_
Resolution range (Å)^a^		50–2.25 (2.33–2.25)	50–2.05 (2.12–2.05)	50–1.90 (1.97–1.90)
Cell dimensions (Å)	a	93.9	67.2	67.3
	b	96.2	96.1	96.0
	c	66.6	102.0	101.6
Total no. of reflections		107,698	270,035	264,815
No. of unique reflections		27,339	42,294	51,836
Completeness (%)^a^		93.5 (96.0)	99.9 (100.0)	98.6 (94.8)
Redundancy^a^		3.9 (3.7)	6.4 (6.0)	5.1 (4.0)
I/σ (I) a		23.9 (4.9)	31.0 (3.4)	22.7 (2.4)
R_merge_ (%)a, b		6.7 (29.6)	7.6 (53.0)	6.6 (43.6)

a Values in parentheses correspond to the shell of highest resolution.

b R_merge_ = ∑_hkl_| I_i_ − I_m_ | / ∑_hkl_<I_m_>, where I_i_ and I_m_ are the observed and mean intensity of related reflections with common indices h, k, and l.

### Structure determination and refinement

We used the crystal structure of VA387 P domain as the search model and the program Phaser [25] to solve the phases of the crystal structure of VA207 P protein. The amino acid sequence was then replaced with that of VA207 P domain, and manual adjustments were carried out with the program COOT [26] by the guidance of (2Fo-Fc) and (Fo-Fc) electron density maps, where Fo is the observed structure factor and Fc is the calculated structure factor. Further adjustment and refinement were carried out with the programs CNS [27], Refmac [28] and Phenix [29]. At the final stage of refinement, water molecules were added in (Fo-Fc) map at peaks (>3σ) where they could form good hydrogen bonds with nearby residues. Statistics for structure refinement are summarized in Table 2. The refined structures were validated with the program PROCHECK [30]. No residue was found in a disallowed region of the Ramachandran plot. The structure analysis was performed by programs EdPDB [31] and PyMOL (DeLano Scientific LLC ).

**Table 2:** Structure refinement statistics.

Parameters	Native P protein	Complex with Le^y^ tetrasaccharide	Complex with SLe^x^ tetrasaccharide
**No. of reflections in working set**	25,018	40,540	48,656
**No. of reflections in test set**	1,284	2,031	2,472
R_work_ a	0.225	0.185	0.186
R_free_ a	0.232	0.204	0.228
**Root mean square deviation**			
Bond lengths (Å)	0.009	0.008	0.008
Bond angles (°)	1.451	1.069	1.146
**Average B factors (Å^2^)**			
Total	32.3	37.0	37.7
Protein	31.8	36.6	36.9
Tetrasaccharide		45.8	55.6
Solvent	38.8	39.7	41.6
**Residues in the Ramachandran plot (%)**			
Favored	97.4	98.7	98.2
Allowed	2.6	1.3	1.8
Disallowed	0.0	0.0	0.0

a R_work_ = ∑| |F_obs_|−|F_cal_| |/∑|F_obs_|, R_free_ = ∑_T_| |F_obs_|−|F_cal_| |/∑_T_|F_obs_|, where F_obs_ and F_cal_ are observed and calculated structure factors, respectively. For R_free_, T is a randomly selected test data set (5.0%) of total reflections and was set aside before structure refinement.

### HBGA-binding assay

The synthetic oligosaccharide-based binding assays were performed basically as described elsewhere [22], [23]. The affinity-column purified P particles were diluted to 10 µg/ml as working solution. Synthetic oligosaccharides representing H1, H2, H3, A, B, Le^a^, Le^b^, Le^x^, and Le^y^ were purchased from GlycoTech (Gaithersburg, MD), while the other two representing SLe^a^ and SLe^x^ were from The Consortium for Functional Glycomics (CFG, USA). The oligosaccharides were coated on 96-well microtiter plates (Dynex Immulon; Dynatech, Franklin, MA). After blocking by nonfat milk, P particles at 10 ng/µl were added. The bound P particle were detected using a rabbit anti-VA207 VLP antiserum (1∶3300), followed by the addition of HRP-conjugated goat anti-rabbit IgG (ICN, Aurora, OH).

### Protein data bank accession

The coordinates and structure factors for the VA207 P protein (3PUM) as well as its complexes with Le^y^ tetrasaccharide (3PUN) and SLe^x^ tetrasaccharide (3PVD) have been deposited in the Protein Data Bank, Research Collaboratory for Structural Bioinformatics, Rutgers University, New Brunswick, NJ.

## Results

### Crystallization of VA207 P protein

Norovirus VA207 (GII.9) has been demonstrated to recognize Le^y^, Le^x^ and SLe^x^ antigens [23] and thus was chosen as a model to study the structure of the binding interface to Lewis antigens. The P domain of VA207 (amino acid 222 to 537) was expressed in *E. coli* and the HBGA binding profile of the resulting P protein was verified (data not shown) before being used in the crystallography study. The protein sample was concentrated to 12 mg/ml and the crystallization was performed in the presence or absence of the Le^y^ or SLe^x^ tetrasaccharide. Structures of the native P protein and the P protein complexed with the oligosaccharide were solved with the molecular replacement method using diffraction data up to 2.2 and 2.0 angstroms (Å) resolution, respectively.

### The overall structure of VA207 P protein protomer

The crystal of the native P protein of VA207 belongs to space group of P2_1_2_1_2 and contains a homodimer of P protein in an asymmetric unit. Most residues from 226 to 527 could be modeled in the electron density map, while residues 295 to 297 and 392 to 394 could not be modeled due to lack of interpretable electron density map in these loop regions. However, in both complex structures with the Le^y^ and SLe^x^ tetrasaccharides, the above missing residues could be modeled, suggesting that binding to either of the two tetrasaccharides stabilizes the two flexible loops.

**Figure 2:**
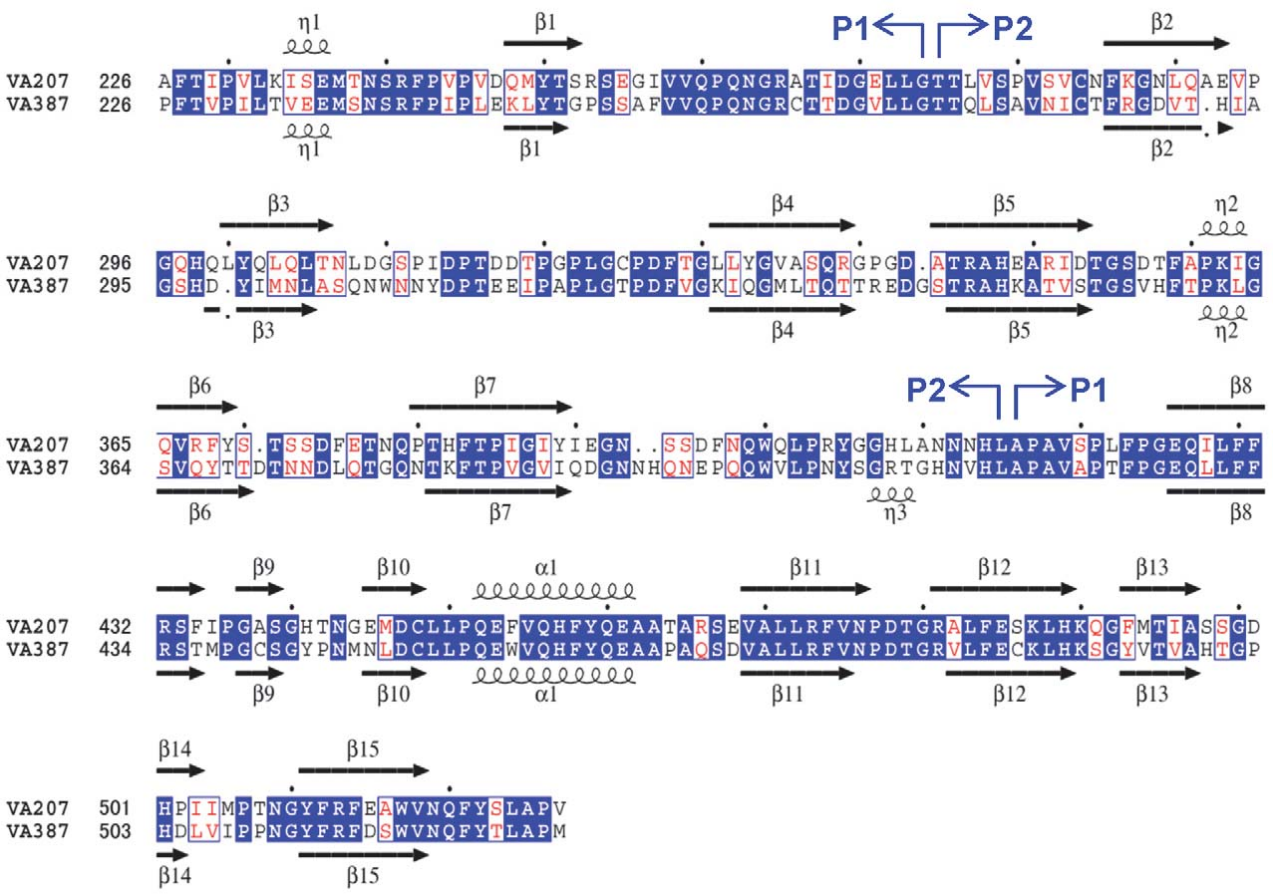
Structure-based sequence alignment between VA207 and VA387 P domains. Boundaries between P1 and P2 subdomains are indicated by blue arrows. Identical residues are highlighted in blue background, while similar residues are shown in red character. The secondary structures that are assigned by the program DSSP are shown for both P domains above and below their respective sequence. Coils represent η helices and α helices, whereas black arrows represent β strands.

**Figure 3:**
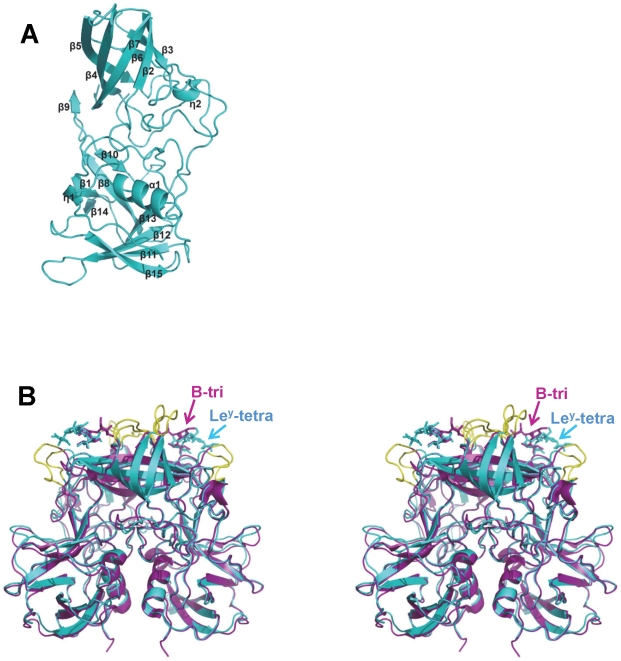
Overall structure of VA207 P protein monomer and comparison of VA207 and VA387 P dimers complexed with oligosaccharides. (A) Ribbon representation of VA207 P monomer shown in cyan. Secondary structural elements are labeled as in Figure 2. (B) Superimposition of the P dimer structures of VA207 (cyan) with Lewis Y tetrasaccharide (Le^y^-tetra) and VA387 (purple; PDB entry: 2OBT) with B trisaccharide (B-tri) in stereo view. The major structural differences in the loop region of P2 subdomain between the two strains are indicated in yellow.

The P protein protomer of VA207 shares similar structure features with that of VA387 (GII.4) [18], [19] and Norwalk virus (GI.1) [4], [18], [20] (Figures 2 and 3). It can also be divided into two subdomains, P1 and P2. P2 subdomain, spanning from residue 275 to 415, is an insertion into P1 subdomain, splitting the latter into two fragments (222 to 274 and 416 to 537, Figure 2). The P1 subdomain has a mixed α/β structure. Two twisted antiparallel β sheets (β1-β8-β10 and β14-β1-β8-β13-β12-β11-β15, where β1 and β8 strands are shared within the two β-sheets) and an α helix form a hydrophobic core (Figure 3). While P1 forms the legs, P2 constitutes the head of the P dimer with a β-barrel made of 6 antiparallel β strands folded as a Greek key topology. The structure of the P protein is very similar between VA207 and VA387, with a perfect match in their P1 subdomains. However, significant differences were observed in the four loops on the P2 subdomain connecting β2-β3, β4-β5, β6-β7 and β7-β8, as a result of sequence variations in the four loops between the two strains (Figure 2).

### Structure of VA207 P protein dimer

The P protein forms a homodimer along a non-crystallographic two-fold axis, and the dimerization is essential for forming a functional carbohydrate binding site on the outermost surface of the virus capsid (Figure 3) (see below). The P dimer has a dimension of 56 by 66 by 70 Å and contains a large buried surface area of 3, 300 Å^2^ (for two protomers) formed by both polar and non-polar residues. Both subdomains contributed to the dimer interface: the α helices in the P1 subdomain interacts with each other by hydrogen bonds and hydrophobic interaction, while strands β5 and β9 from the β barrel in P2 subdomain was bound mainly by hydrogen bonds. Similar to the protomers, the general structure of VA207 P dimer is very similar to that of VA387, particularly in the P1 subdomain region. However, clear difference can be seen on the top surface of the P dimer (Figures 3).

### The carbohydrate-binding interface of VA207

**Figure 4:**
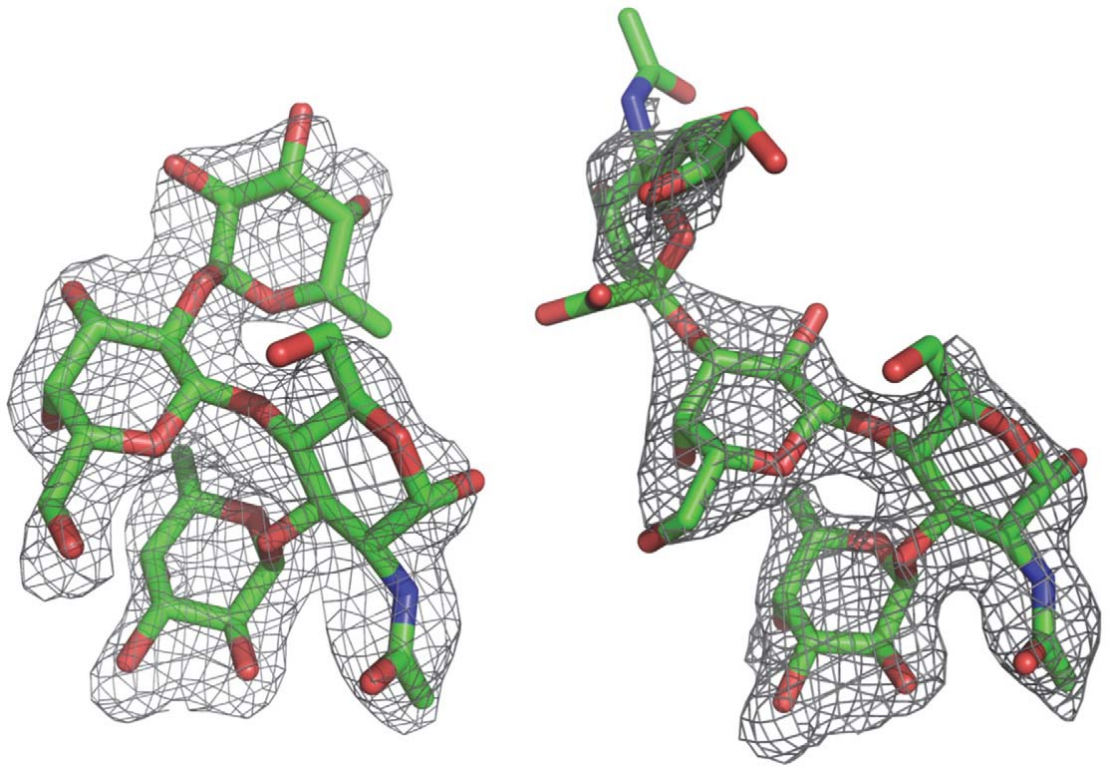
(Fo-Fc) omit electron density map of the Lewis y and 3′-sialyl Lewis x tetrasaccharide. The map was calculated with the final structures of P protein coordinate without corresponding tetrasaccharides and was contoured at 2.0σ (grey) with a 1.6Å radius coverage. Carbon, oxygen and nitrogen atoms are in green, red, and blue, respectively.

Co-crystallization of the P protein with Le^y^ and SLe^x^ tetrasaccharides has resulted in crystals in a new form of space group P2_1_2_1_2_1_ instead of the P2_1_2_1_2 native form. In this new crystal form, an asymmetric unit also contains one homodimer along a noncrystallographic two-fold axis. The two complex structures were solved with the molecular replacement method using the native P protein dimer structure as the initial model. In both complex crystals the four saccharide rings could be clearly discerned from the (fo-fc) electron density map at a resolution of 2Å, and their structures were modeled accordingly (Figure 4). Torsion angles of glycosidic linkages for the two tetrasaccharides are listed in Table 3.

**Table 3:** Torsion angles of glycosidic linkages in Le^y^ and SLe^x^.

tetrasaccharides	saccharides	phi(Φ)	Psi(ψ)
Le^y^	α-1,3 Fuc	−83.2(−83.4)^a^	−103.8(−94.1)
	β-1,4 Gal	−71.6(−62.4)	132.9(128.4)
	α-1,2 Fuc	−80.8(−79.9)	131.3(122.8)
SLe^x^	α-1,3 Fuc	−80.4(−87.6)	−104.7(−99.1)
	β-1,4 Gal	−73.6(−72.8)	140.9(135.9)
	Sialyl acid	60.3(49.3)	−136.4(−125.4)

a Values in parentheses correspond to the tetrasaccharide at the other binding pocket related by a non-crystallographic two fold axis in the dimer structure.

**Figure 5:**
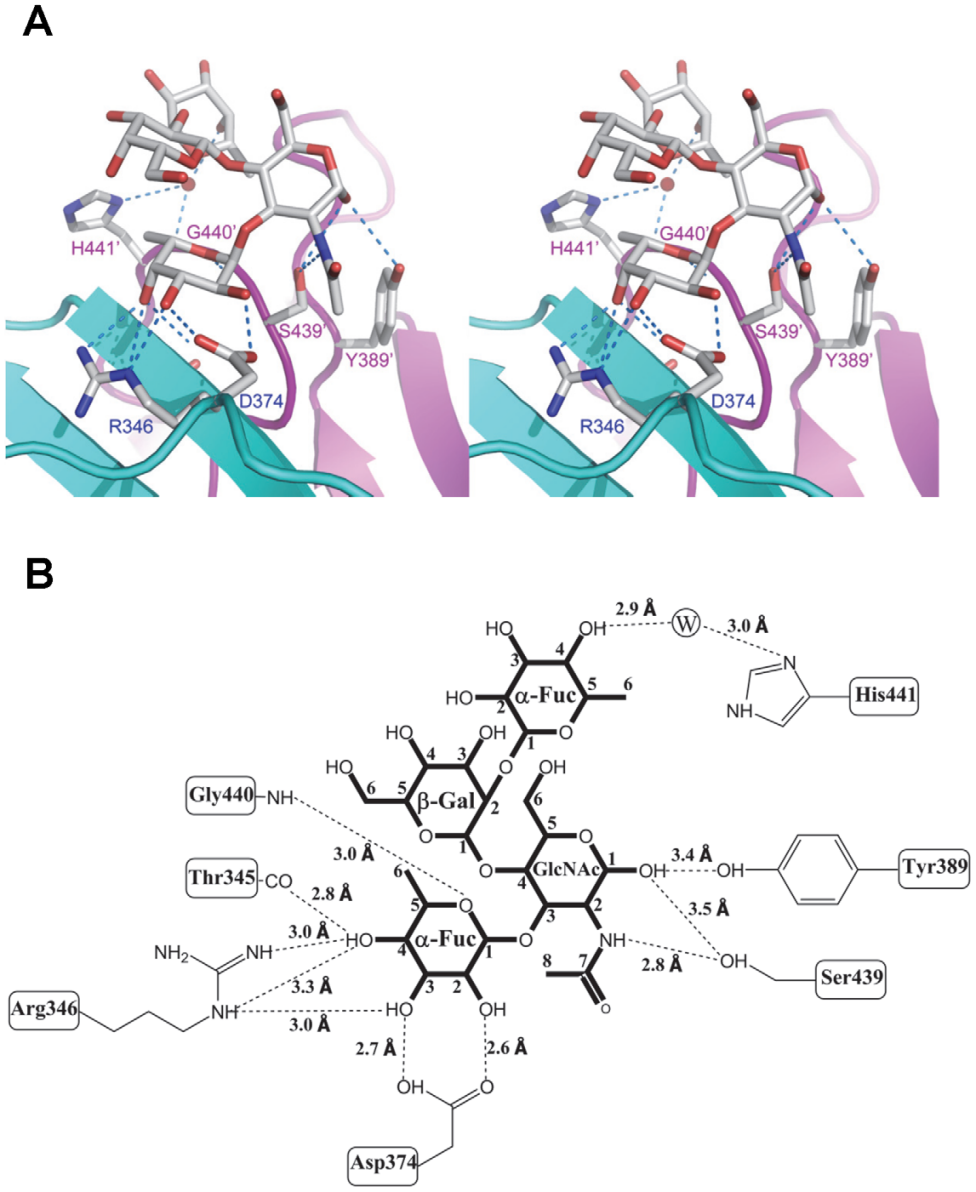
Extensive interaction network between VA207 P dimer and Lewis Y tetrasaccharide. (A) Stereo view of the binding cavity of VA207 P dimer and the bound Lewis Y tetrasaccharide. Protein structure is in ribbon representation as in Figure 3, while tetrasaccharide and major amino acid residues involved in interaction are in stick style. Hydrogen bonds are indicated by blue dotted lines; carbon, oxygen and nitrogen atoms are in grey, red and blue, respectively. The two P protomers are colored in cyan and purple and residues involved in saccharide interactions are labeled. (B) Schematic diagram showing P protein and Lewis Y tetrasaccharide interaction. Residues of P protein involved in saccharide binding are labeled with detailed atomic information. Hydrogen bonds are shown as dashed lines with indication of H-bond lengths.

**Figure 6:**
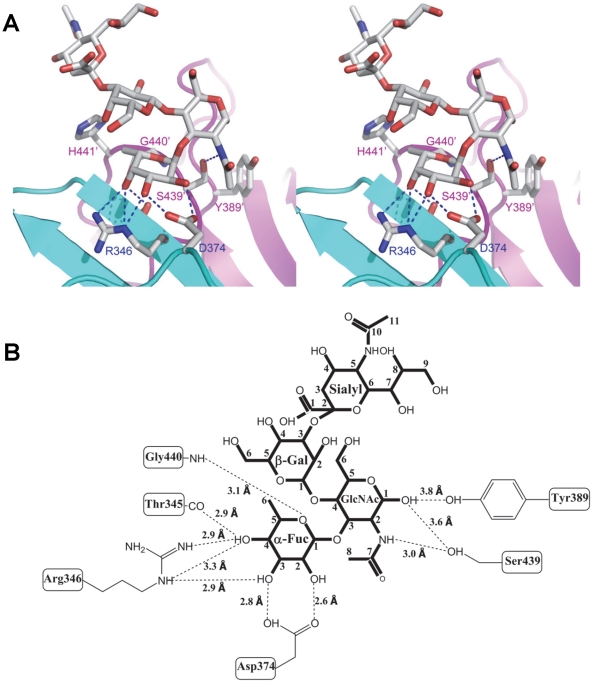
Extensive interaction network between VA207 P dimer and 3′ sialyl-Lewis X tetrasaccharide. (A) Stereo view of the binding cavity of VA207 P dimer and the bound 3′ sialyl-Lewis X tetrasaccharide. Protein structure is in ribbon representation, while tetrasaccharide and major amino acid residues involved in interaction are in stick style. Hydrogen bonds are indicated by blue dotted lines; carbon, oxygen and nitrogen atoms are in grey, red and blue, respectively. The two P protomers are in cyan and purple and residues involved in saccharide interactions are labeled. (B) Schematic diagram of P protein and 3′ sialyl-Lewis X tetrasaccharide interaction. Residues of P protein involved in the interaction are labeled with detailed atomic information. Hydrogen bonds were shown as dashed lines with indication of H-bond lengths.

**Figure 7:**
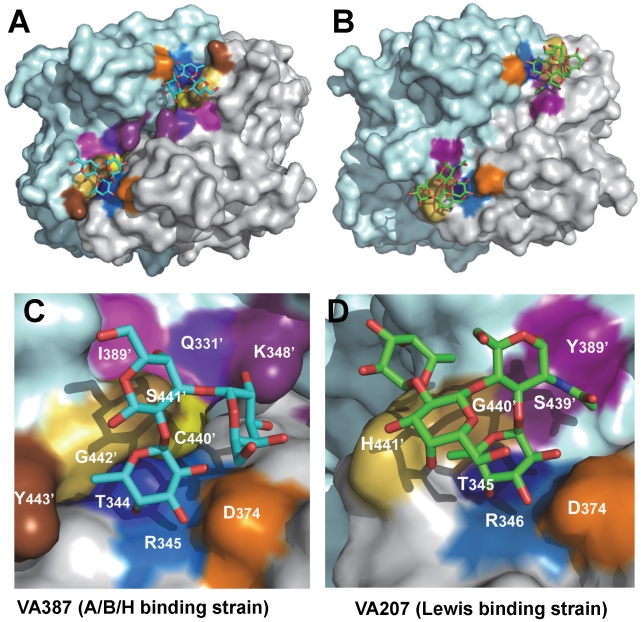
The crystal structures of the HBGA-binding interfaces of VA387 (GII.4) and VA207 (GII.9). (A and B) The surface models of the P dimers (top views) of VA387 (A) and VA207 (B). One protomer is shown in light cyan, while the other in grey. The HBGA-binding interfaces are shown in darker colors. (C and D) Enlargements of the HBGA-binding interfaces with labels of individual amino acids. The prime symbol indicates a residue of the other protomer. The three major components of the binding pocket are colored in blue (bottom), orange (wall), and yellow/sand (another wall), respectively, while the nearby site that affects specificity of the binding interface is shown in purple. The type B trisaccharide binding to VA387 (A and C) is shown in cyan (C), red (O), and blue (N), while the Le^y^ tetrasaccharide binding to VA207 (B and D) is shown in green (C), red (O), and blue (N).

The carbohydrate binding site of VA207 is located at the distal surface of the P dimer (Figure 3), corresponding to the outermost surface of a norovirus capsid. Like VA387 [19] the ligand binding site is located in the interface between two P proteins, indicating both protomers contribute to the ligand binding. The binding pocket for the Le^y^ and SLe^x^ tetrasaccharide is constituted by 7 amino acid residues, of which T345, R346, and D374 are from one protomer forming the “bottom” (T345 and R346) and a “wall” (D374), while G440, H441, S439 and Y389 are from the other protomer constituting the other “walls” of the pocket (Figures 5, 6 and 7).

Extensive hydrogen bond networks were observed between these amino acid residues and the tetrasaccharides of the Lewis antigens. In particular, T345, R346, D374 and G440 interact with the α-1, 3 fucose (Lewis epitope), Y389 and S439 with the N-acetylglucosamine saccharide, one of the two precursor saccharides, and H441 with the α-1, 2 fucose (H epitope on Le^y^ tetrasaccharide) via a water molecule as a bridge (Figures 5 and 6). It is noted that 5 of the 7 amino acid residues that participate directly in interaction of VA207 with the Le^y^ antigen are conserved in VA387 (GII.4) [11], [19], supporting the notion that these two GII strains share a conserved genogroup II HBGA-binding interface as predicted previously [11]. However, unlike VA387 that recognizes A and B antigens through the α-1, 2 fucose (H epitope) as its major contact [19], VA207 interacts with the two Lewis antigens through the α-1, 3 fucose (Lewis epitope) as its main recognition moiety. Thus, a conserved carbohydrate binding interface can recognize different HBGAs with different modes among genetically related but distinct GII viruses.

### The sialic acid did not participate in binding with VA207

**Figure 8:**
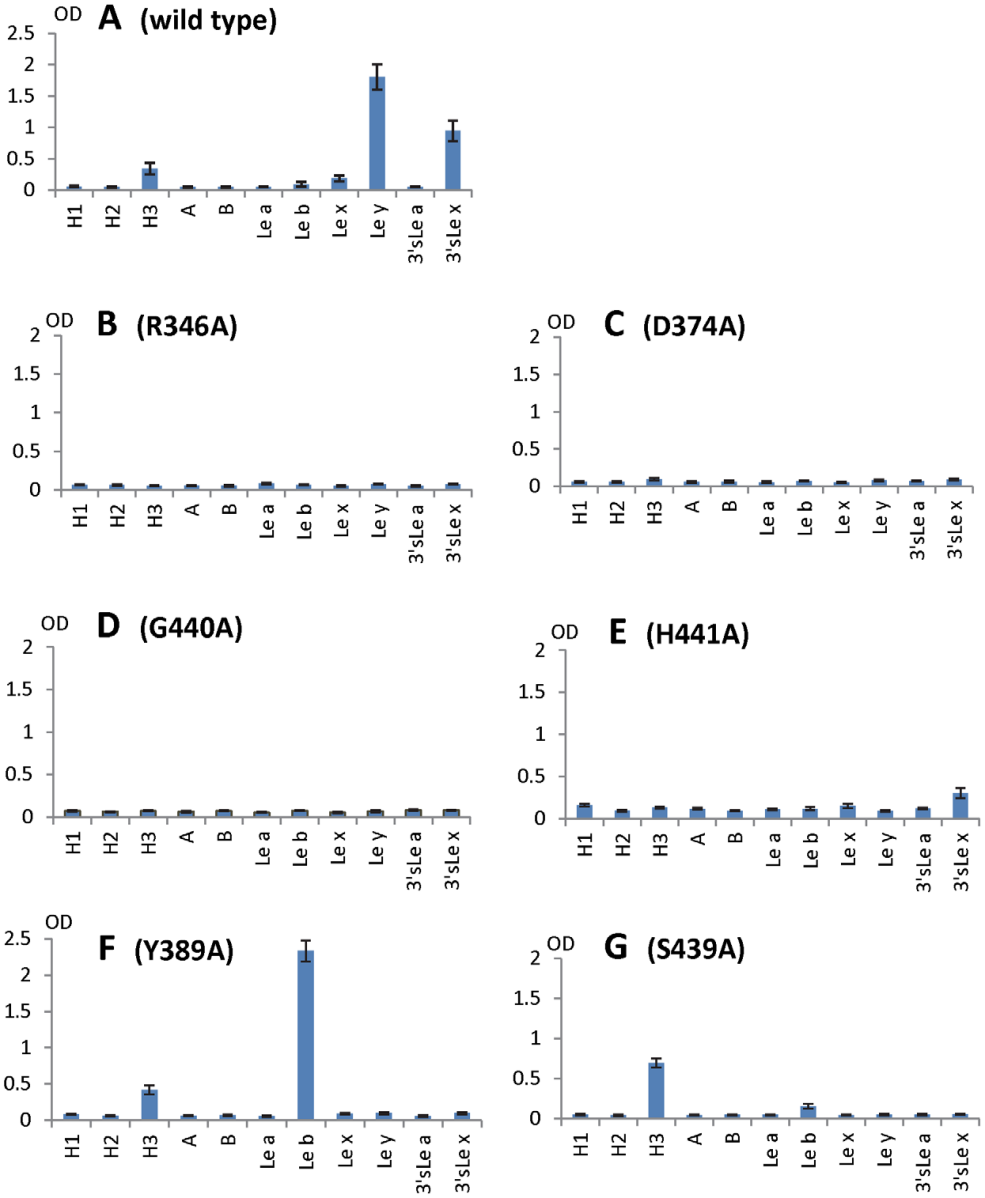
HBGA-binding outcomes of various mutant P particles with single amino acid mutations in the HBGA-binding interface of VA207. Binding of wild type (A) and mutant P particles (B to G) to a panel of synthetic oligosaccharides representing HBGAs of H1, H2, H3, A, B, Le^a^, Le^b^, Le^x^, Le^y^, 3′sLe^a^, and 3′sLe^x^ (X-axes). Y axes indicate the optical densities at 450 nm (OD_450_) that were the average values of quintuplicate (A) and triplicate (B to G) experiments, respectively. Concentrations of the P particles were 10 µg/ml.

The sialyl modified Le^x^ was recently suggested to participate in binding to some GII noroviruses [32], [33], and our binding assays showed that VA207 binds strongly to the SLe^x^ (Figure 8A). Therefore, we particularly studied whether the sialic acid residue participates in binding of VA207 by co-crystallization of the P protein with SLe^x^. Our data showed the sialic acid was far away from the P dimer surface (Figure 6) and there seemed no hydrogen bond or other direct interaction with the VA207 capsid. The number of hydrogen bonds between the SLe^x^ and P dimer was reduced to 9, which is one less than that in P dimer-Le^y^ complex, due to the absence of the α-1, 2 fucose in the SLe^x^ tetrasaccharide (Figures 5 and 6).

### Both α-1, 3 and α-1, 2 fucoses are critical for interacting of VA207 with HBGAs

To further characterize the role of individual amino acids in the HBGA-binding interface, mutations were introduced to residues that form hydrogen bonds with carbohydrate ligands followed by binding assays of the mutant P particles [7], [8] to HBGA receptors. When single amino acid mutations were introduced to amino acids interacting with the α-1, 3 fucose- (R346A, D374A and G440A) and the α-1, 2 fucose (H441A) (Figures 5 and 6), all mutant P particles lost their binding completely (R346A, D374A and G440A) or nearly completely (H441A) to H3, Le^y^, Le^x^ and SLe^x^ compared with the wild type P particles (Figure 8 A to E). Saliva-based binding assay confirmed the results (data not shown) [11]. These data indicated that both α-1, 3 and α-1, 2 fucoses are important in VA207 recognition of HBGAs, in which the interactions of the α-1, 3 fucose may be more important.

### The N-acetylglucosamine binding site affects the binding specificity of VA207

**Figure 9:**
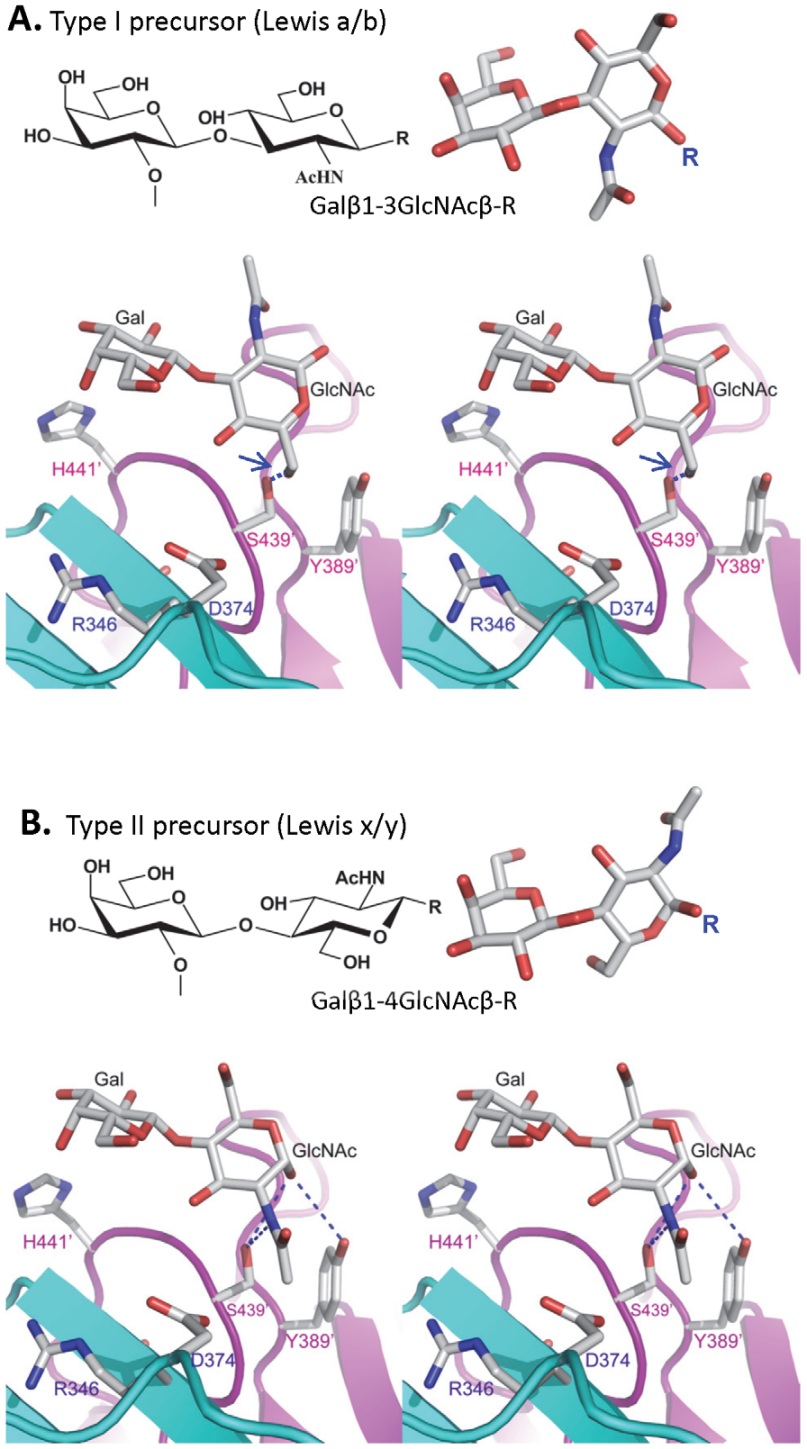
The structures of the type I and type II disaccharide precursors of HBGAs and their possible steric locations interacting with the VA207 P dimer. The type I (A) and type II (B) disaccharide precursors are shown in stereo view. The two protomers are shown in cyan and purple, respectively and atoms are colored in grey (C), red (O), and blue (N). The –CH3OH group of the GlcNAc of the type I disaccharide collides (1.3 Å) with the side chain of S439 (A, right, marked by a blue arrow), making the Le^b^ sterically difficult to bind to the wild type VA207. R indicates the position that links to backbone. Blue dashed lines in panel B indicate the hydrogen bonds between GlcNAc and Y389/S439 in complex structure. The two fucose residues representing H and Lewis epitopes are omitted for clarity. Gal, galactose; GlcNAc, N-acetylglucosamine.

When single mutations were introduced to the two amino acids that form the N-acetylglucosamine (GlcNAc) binding site (S439A and Y389A) observed for VA207, the resulting VA207 P particle mutants demonstrated a change in binding specificity to HBGAs (Figure 8, comparing A with F and G). While both mutants lost binding to the Le^y^ and SLe^x^, the Y389A mutant gained a strong binding to Le^b^ (Figure 8F), and the S439A mutant retained binding to H type 3 antigen (Figure 8G). Since the GlcNAc of Le^b^, Le^y^/Le^x^, and H type 3 are from type I, II, and III precursors, respectively, with different linkages and side chains (Figure 9), our data indicated that this GlcNAc binding site determines the specificity of VA207 in a type-specific manner (see discussion).

## Discussion

In this study we determined the atomic structure of the carbohydrate binding interface of the first Lewis-binding norovirus (VA207, GII.9) using X-ray crystallography followed by a mutagenesis study. As a member of GII noroviruses, VA207 is expected to share a conserved carbohydrate binding interface [11] with that of VA387 (GII.4) [10], [19]. This has been now demonstrated in this study by showing that the HBGA binding sites of VA207 and VA387 share highly conserved location, amino acid composition and overall structures of the binding pockets. We also elucidated the structural basis of the two strains in recognizing different sets of HBGAs through different interaction modes. VA207 recognizes the Lewis antigens through the α-1, 3 fucose (the Lewis epitope) as a major contact (Figures 5 and 6), while VA387 interacts with the secretor antigens through the α-1, 2 fucose (the H epitope) as the major binding target [10], [19]. These data provide structural evidence on how subtle changes in the HBGA binding interface affect binding specificity and revolution of genetic linkage of noroviruses under the selection by the polymorphic human HBGAs.

Genetically, VA207 and VA387 belong to two genotypes within the same genogroup II but share only 54% amino acid identity in the P domains. However, the P dimers of the two strains share very similar structures with a nearly perfect match between their P1 subdomains. The basic structures of the carbohydrate binding pockets of the two strains are also highly conserved, in which 4 of the 5 amino acids constituting the core structure of the binding pockets are identical between the two strains. These data indicate that the recognition of HBGAs is a prerequisite of norovirus infection, which plays an important role in norovirus evolution. In contrast, no such similarity is found with Norwalk virus (GI.1), confirming the genetic linkage between the two GII noroviruses. Major sequence variations between these two GII noroviruses have also been found at the top surface (Figure 7, top panel) of the P2 subdomains. This region is associated with the host immune selection in addition to HBGA recognition.

The conservation of the HBGA binding sites raises a question on how the strain-specificities of HBGA binding are determined. VA207 binds the Le^x^, Le^y^ and H type 3 antigens, while VA387 has a broader binding spectrum to A, B, H type 3, Le^b^, and Le^y^ [22], [23]. Our mutagenesis analysis on both VA387 [10] and VA207 (this report) indicated that the integrity of the core structure of the HBGA binding pocket is strictly required for the binding function. Introducing single mutations to any residue involved in the core structures have resulted in completely loss of binding to all HBGA types ([10], Figure 8 B to E). These core structures include those interacting to the α-1, 3 fucose of VA207 (this report), the α-1, 2 fucose of VA387 and Norwalk virus, and the GalNAc of Norwalk virus [10], [11], [18], [19], [20].

However, some residues outside the core structures also play a role in determining the binding specificity of noroviruses and mutations at these residues can change carbohydrate targets depending on the types of HBGAs being involved. For example, Q331 and K348 in VA387 interact with GalNAc of the A antigen and α-Gal of the B antigen [10], [11], while S439 and Y389 in VA207 interact with GlcNAc (Figures 4 and 5), a β-1, 4 linked saccharide in the type II precursor of Le^x^ and Le^y^ antigens. These residues are located in the similar positions outside the core structures of the binding pockets of the two GII strains (Figure 7), providing further evidence of selection of noroviruses by the host HBGAs. The demonstration of carbohydrate target switch helps the understanding of the complex interactions between the diverse noroviruses and polymorphic HBGAs of human hosts.

Additional strain-specificity of VA207 binding to HBGAs was observed through the mutagenesis study of S439 and Y389. Several hydrogen bonds between the GlcNAc of Le^y^ and SLe^x^ and the side chains of S439 and Y389 were evident in crystallography studies (Figures 5 and 6). These bonds, together with those with the α-1, 3 and the α-1, 2 fucoses, form an interacting network responsible for the binding of VA207 to the Le^y^ and the SLe^x^ antigens. A single mutation of Y389A lost the binding to the type II Lewis antigens (Le^x^, SLe^x^, and Le^y^) but gained strong binding to the type I Lewis antigen (Le^b^), while no change in binding to the H type III antigens (Figure 8, comparing A with F). Similarly, the S439A mutant abolished the binding to Le^x^ and Le^y^ without altering the binding to H type III (Figure 8, comparing A with G). These results can be well explained by the structural difference between the type I (Le^a^ and Le^b^) and type II (Le^x^ and Le^y^) Lewis antigens. Both Le^x^ and Le^y^ are derivatives of the type II precursor (Galβ1-4GlcNAcβ-R, Figure 9B), of which the β-1, 4 GlcNAc interacts with S439/Y389 (Figures 5, 6, and 9B), whereas the Le^b^ is a derivative of the type I precursor (Galβ1-3GlcNAcβ-R, Figure 9A) with a β-1, 3 GlcNAc. Thus, in Le^b^, the glycosidic linkages of the GlcNAc with α-Fuc and β-Gal should be switched compared with Le^y^, resulting in the GlcNAc residue flipping by about 180°around the axis which is across and perpendicular to the C1-O5 and C3-C4 bonds of the GlcNAc. Consequently, the –CH3OH group of the GlcNAc in Le^b^ would collide with the side chain of S439 (1.3 Å, Figure 9A, right), making the Le^b^ sterically difficult to bind to the wild type VA207. However, the explanation as to how the two mutations (Y389A and S439A) changed the binding specificity of VA207 P protein remains unidentified based on the current complex structures. Since a simple replacement of the Y389 and S439 with an alanine in the crystal structure cannot explain well the observed change of binding specificity, unknown conformational change in the vicinity of Y389 and S439 may occur due to the mutation, which remains to be defined. These data indicate that the precursor may be a determinant of the type-specific HBGA interaction of noroviruses.

The facts that VA207 binds strongly to Le^y^ but weakly to Le^x^ and that the Y389A mutant binds strongly to Le^b^ but not to Le^a^ suggest that the interaction between H441 and α-1, 2 fucose may be important for binding activity. H441 forms a water-mediated hydrogen bond with α-1, 2 fucose of Le^y^ (Figure 5), most likely also of Le^b^ in the Y389A mutant, which was absent in either Le^a^ or Le^x^; this may be the reason of difference of binding between Le^a^/Le^x^ and Le^b^/Le^y^. It may also explain why the H441A mutation abolishes all binding function. In addition, we noticed the presence of Van der Waals interaction between the side chain of H441 and 6-methyl group of the α-1, 3 fucose (Lewis epitope), which further emphasizes the importance of the H441 in the binding function of VA207. A similar structure formed by Y443 is also present in VA387 which interacts through Van der Waals force with the α-1, 2 fucose of the A/B antigens, and this hydrophobic interaction has been shown to be vital for the binding to HBGA receptors [19].

The role of sialic acid in binding of SLe^x^ to VA207 was suggested by our *in vitro* binding assays (Figure 8A). Similar binding activities of SLe^x^ to GII.4 viruses were also observed in two previous studies [32], [33]. However, our crystallography data excluded a direct participation of the sialic acid in binding to VA207 by the lack of direct interaction between the sialic acid and the P protein in the crystal structure. We hypothesize that the addition of the sialic acid residue may stabilize the structure of the SLe^x^ antigen and thus impact positively the binding to VA207 compared with Le^x^ without sialic acid. Since VA387 is the only GII.4 virus with known crystal structure of the HBGA binding interface, we measured whether VA387 binds to SLe^a^ and SLe^x^ antigens. We did not observe binding of VA387 to either antigens. We also noticed weaker interactions of VA207 wild type and mutants with Le^x^ and Le^a^ than Le^b^ and Le^y^ (Figure 8), in which the occurrence of the α-1, 2 fucose (H epitope) in the Le^b^ and Le^y^ antigens clearly play a role. Further study is needed to clarify this issue.

The binding of VA207 to the H type 3 (Fucα1-2Galβ1-3GalNAc-R1, H3) antigen in addition to the two Lewis antigens (Le^x^ and Le^y^) extends our understanding on flexibility of carbohydrate binding mode. Since the H antigen does not have an α-1, 3 fucose (Lewis epitope, [3], [14]), VA207 must recognize another saccharide as the major contact. The fact that single mutations at the core binding pocket abolish binding to all HBGAs indicates that VA207 must recognize the H type 3 antigen through the same binding interface. However, mutations of S439A and Y389A did not decrease the binding to H type 3 antigen, suggesting that this GlcNAc binding site is not required for binding to the H type 3. Thus, future study would be of significance to find out how the same binding interface of VA207 binds to the H type 3 antigen with a different binding mode in addition to the known binding to Le^x^ and Le^y^ antigens.

In summary, our study elucidates the structural basis of norovirus-Lewis antigen interaction at atomic resolution, which well explains the type-specific HBGA recognition. The structural and functional data generated in this study are valuable to understanding the complex interaction between diverse noroviruses and the polymorphic HBGAs of human hosts. Our current results also highlight the importance of human HBGAs as a critical factor in norovirus evolution, in which a functional carbohydrate binding interface is a prerequisite for norovirus survival. The high resolution pictures of the Lewis antigen binding interface illustrated in this study expand the foundation of our strategies to control and prevent norovirus-associated diseases.
